# Treatment of experimental adjuvant arthritis with a novel folate receptor-targeted folic acid-aminopterin conjugate

**DOI:** 10.1186/ar3304

**Published:** 2011-04-04

**Authors:** Yingjuan Lu, Torian W Stinnette, Elaine Westrick, Patrick J Klein, Mark A Gehrke, Vicky A Cross, Iontcho R Vlahov, Philip S Low, Christopher P Leamon

**Affiliations:** 1 Endocyte, Inc., 3000 Kent Avenue, West Lafayette, IN 47906, USA; 2 Department of Chemistry, 560 Oval Drive, Purdue University, West Lafayette, IN 47907, USA

## Abstract

**Introduction:**

Folate receptor (FR)-expressing macrophages have been shown to accumulate at sites of inflammation, where they promote development of inflammatory symptoms. To target such a macrophage population, we designed and evaluated the biologic activity of EC0746, a novel folic acid conjugate of the highly potent antifolate, aminopterin.

**Methods:**

Using a FR-positive subclone of murine macrophage-derived RAW264.7 cells and rat thioglycollate-elicited macrophages, we studied the effect of EC0746 on dihydrofolate reductase activity, cell proliferation, and cellular response towards bacterial lipopolysaccharide as well as IFNγ activation. The EC0746 anti-inflammatory activity, pharmacokinetics, and toxicity were also evaluated in normal rats or in rats with adjuvant-induced arthritis; that is, a FR-positive macrophage model that closely resembles rheumatoid arthritis in humans.

**Results:**

EC0746 suppresses the proliferation of RAW264.7 cells and prevents the ability of nonproliferating rat macrophages to respond to inflammatory stimuli. In the macrophage-rich rat arthritis model, brief treatment with subcutaneously administered EC0746 is shown to mediate an FR-specific anti-inflammatory response that is more potent than either orally administered methotrexate or subcutaneously delivered etanercept. More importantly, EC0746 therapy is also shown to be ~40-fold less toxic than unmodified aminopterin, with fewer bone marrow and gastrointestinal problems.

**Conclusions:**

EC0746 is the first high FR-binding dihydrofolate reductase inhibitor that demonstrates FR-specific anti-inflammatory activities both *in vitro *and *in vivo*. Our data reveal that a relatively toxic anti-inflammatory drug, such as aminopterin, can be targeted with folic acid to inflammatory macrophages and thereby relieve inflammatory symptoms with greatly reduced toxicity.

## Introduction

A phenomenon characteristic of many autoimmune and inflammatory disorders is persistent and unrestrained macrophage activation [1]. This extensive build-up of tissue-infiltrating macrophages consists of a destructive cell population made up of both locally activated macrophages and inflammatory monocytes that have been recruited from the blood in large quantities. In rheumatoid arthritis (RA) the synovial joints are enriched with these activated macrophages, where they play a primary role in the pathophysiology of joint destruction and disease progression [2,3]. Based on the concept that inflammatory diseases can be caused or worsened by activated macrophages, many therapeutic interventions for inflammatory disorders have focused on suppressing or neutralizing one or more proinflammatory products released by these macrophages. Examples of such therapeutics include agents that reduce TNFα (for example, etanercept, infliximab, adalimumab), IL-1 (anakinra), and IL-6 (tocilizumab, atlizumab) [4,5]. Other biologic agents targeting IL-12/IL-23 (ustekinumab), B cells (rituximab), and T cells (abatacept, alefacept) are also available as a second-line or third-line treatment when anti-TNF agents fail [6]. Despite remarkable success, biologics remain prohibitively expensive (~$16,000 per year) [7], and a majority of them carry a black-box warning for increased risk of serious infections [8].

Alternatively, methotrexate (MTX; molecular weight 454.4) has a long history of use in treating rheumatic diseases, and it continues to be the most prescribed medicine (taken orally at 7.5 to 25 mg/week) even in the current era of the aforementioned biologic therapies [9]. As a well-known antifolate, MTX inhibits multiple folate-dependent enzymes involved in biosynthesis of purines/thymidylate and several amino acids, in particular dihydrofolate reductase (DHFR) and 5-aminoimadazole-4-carboxamide ribonucleoside transformylase [10,11]. Inhibition of 5-aminoimadazole-4-carboxamide ribonucleoside transformylase also causes the release of adenosine, a potent endogenous anti-inflammatory agent, at sites of inflammation [11]. Although the precise anti-inflammatory mechanism(s) by which MTX functions remains unclear, its therapeutic activities may include suppression of proliferation of immune cells responsible for inflammation, induction of T-cell apoptosis, and alterations in cell recruitment and cytokine production [11]. Compared with most disease-modifying anti-rheumatic drugs, MTX is generally considered to be well tolerated, and people who are prescribed MTX can remain on this medication for many years [9]. Approximately one-third of RA patients discontinue MTX therapy, however, due to drug-related toxicities and/or poor responses [12,13], and many are put on a combination treatment with a biological agent [9].

The molecular predecessor of MTX is aminopterin (AMT), a compound that was initially discovered as a chemotherapeutic agent but was abandoned in the 1950s in favor of MTX due to high toxicity and low therapeutic index [14]. Historically, AMT was the first antifolate used to treat inflammatory disorders (RA and psoriasis), and it produced a rapid improvement in disease activity, but not without toxic reactions [14]. There is renewed interest in AMT, however, because while it shares similar pharmacological actions to MTX, the predecessor appears to be more potent when compared in murine models of air-pouch inflammation (~40-fold) and arthritis (~20-fold) [15,16]. The superior anti-inflammatory action of AMT is due in part to its higher affinity for folypolyglutamate synthetase, an enzyme responsible for intracellular retention of folates and antifolates [15,17]. In preclinical studies, however, the increased potency of AMT does not come without an increase in toxicity, wherein the reported acute 50% lethal dose for oral exposure of AMT in mice is 3 mg/kg, compared with 89 mg/kg for MTX [18]. A contributing factor to this toxicity is that, like MTX and most antifolates, AMT enters cells via the reduced-folate carrier (RFC), a ubiquitously expressed anion transporter present in normal tissues [19], and probably through a second ubiquitously expressed proton-coupled folate transporter that is responsible for intestinal folate resorption under low pH conditions [20].

While clinical success of antifolates for the treatment of inflammatory diseases validates the pharmacology of this class of agents, the issues of toxicity and temporal response highlight the need for further improvement. One possible solution for decreasing the toxicity due to nonspecific exposure while at the same time enhancing drug uptake at the inflamed site is to target the antifolate more selectively to the inflammatory cells of interest. A potential cellular target for increased selectivity in the treatment of inflammatory diseases came with the discovery that activated (but not resting) macrophages express a functional folate receptor (FR) known as FRβ [21-23]. This finding has allowed for the rational exploitation of FR-mediated therapeutic intervention as well as diagnostic tools (reviewed in [24,25]). For example, folate-targeted imaging agents have been shown to accumulate in sites of active inflammation in animals [25,26] and selectively within the inflamed joints of RA patients [27]. Likewise, recent efforts in therapy include dsFv anti-FRβ-targeted *Pseudomonas *exotoxin A [28], folate-hapten-mediated immunotherapies [29,30] and antifolates designed to bind FR [23,31]. Although each of the aforementioned approaches holds promise for yielding new therapeutic options for patients, there have been no reports to date on the use of folic acid (FA) for targeting small molecular weight anti-inflammatory drugs to sites of inflammation.

In our present study we investigated the biological activities of EC0746, a FA-AMT conjugate designed to intracellularly deliver an AMT analog specifically via the FR. The anti-inflammatory activity of EC0746 was evaluated in a series of *in vitro *and *in vivo *studies using FR-positive macrophage cell lines and a rat adjuvant-induced arthritis (AIA) model. Since MTX and etanercept are part of the current standard of care for RA and other rheumatic diseases, EC0746 was also compared against oral MTX at equimolar doses and against a limited high-dose regimen of etanercept. Finally, we determined the plasma pharmacokinetics and the maximum tolerated dose (MTD) of EC0746 versus AMT. Our investigation provides the first evidence that a FA-targeted small molecule anti-inflammatory agent may be useful as a macrophage-specific intervention with an advantage of improved therapeutic index over its parent drug.

## Materials and methods

### Reagents

**Figure 1:**
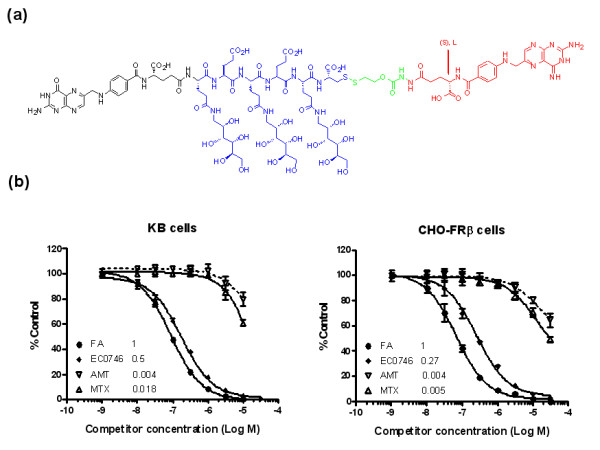
**EC0746 folate receptor binding affinities**. **(**a**) **Chemical structure of EC0746. There are four separate functional components to this novel construct: the folate receptor (FR)-targeting moiety folic acid (FA; black), the drug moiety aminopterin (AMT; red), a saccharo-amino acid peptide-based spacer of ((saccharo-γGlu)-γGlu)_2_-γCys (blue), and a hydrazide/disulfide-containing linker (green). **(**b**)** Relative binding affinities of EC0746 in comparison with AMT and methotrexate (MTX) using FRα-expressing KB cells and FRβ-expressing CHO-FRβ cells. The assays were performed in triplicate at 37°C using each compound as a competitor to displace [^3^H]FA from binding to FR-expressing cells. Numbers shown next to each test article are relative affinity values with FA itself set at 1.

EC0746 (molecular weight 2,236), shown in Figure 1a, was synthesized as previously described [32]. [^3^H]FA was purchased from Amersham (Arlington Heights, NY, USA). AMT, MTX, *Pseudomonas aeruginosa *lipopolysaccharide (LPS), and a DHFR colorimetric assay kit were purchased from Sigma-Aldrich (St Louis, MO, USA). Murine IFNγ was purchased from PeproTech (Rocky Hill, NJ, USA). Etanercept was purchased from CVS Pharmacy (West Lafayette, IN, USA). The cell proliferation (2,3-bis(2-methoxy-4-nitro-5-sulfo-phenyl)-2H-tetrazolium-5-carboxanilide (XTT)) and TNFα ELISA kits were purchased from Roche Applied Science (Indianapolis, IN, USA) and eBioscience (San Diego, CA, USA), respectively. The Proteome Profiler™ rat cytokine array (Panel A) was purchased from R&D Systems (Minneapolis, MN, USA). Heat-killed *Mycobacteria butyricum *was purchased from BD Diagnostic Systems (Sparks, MD, USA). All other reagents were obtained from major suppliers.

### Animals, thioglycollate-elicited peritoneal macrophages, and cell culture

All animal care and use were performed according to National Institutes of Health guidelines and in compliance with protocols approved by the Purdue Animal Use and Care Committee. Female Lewis rats (175 to 200 g) were purchased from Harlan Sprague Dawley (Indianapolis, IN, USA) and were allowed to acclimate for 1 week. To obtain peritoneal macrophages, rats were dosed intraperitoneally with an aged thioglycollate (TG) medium (20 ml/kg) and euthanized 3 days later. The peritoneal cavity of the animals was lavaged with 60 ml ice-cold phosphate-buffered saline to collect peritoneal exudate. The TG-elicited macrophages in the peritoneal fluids were obtained after a red cell lysing step and a 2-hour adherence in folate-free RPMI 1640 medium (Mediatech, Manassas, VA, USA) containing 1% heat-activated fetal calf serum and antibiotics. The purity of the resulting macrophage population (herein referred to as rat TG-macs) was determined to be ~90% pure based on CD11b/c expression (data not shown). The RAW264.7 macrophage cell line is a FR-expressing subclone of ATCC TIB71 (a murine macrophage-derived tumor cell line) that has been adapted to grow under folate-deficient conditions. Previously we have reported that rat TG-macs express ~20-fold less FR than RAW264.7 cells, but these receptors (isotope not identified due to the lack of anti-rat FRβ antibodies) can internalize folate conjugates at a rate consistent with their levels of FR expression [25]. Unless otherwise specified, all cells were maintained in the folate-free RPMI 1640 medium containing 10% heat-activated fetal calf serum and antibiotics (FFRPMI) under a 5% CO_2 _atmosphere.

### Relative affinity assays

The relative affinities of EC0746, AMT, and MTX were determined according to a previously established method except that both KB cells and CHO-FRβ cells were used as the sources of FR [33]. KB cells are a human cancer cell line known for elevated expression of FRα. CHO-FRβ cells were obtained from Manohar Ratnam, Department of Biochemistry and Cancer Biology, The University of Toledo (Toledo, OH, USA). This cell line was originally generated by stable integration and amplification of a human FRβ cDNA expression construct in CHO-K1 cells [34]. The relative affinity value was defined as the inverse molar ratio of compound required to displace 50% of [^3^H]FA bound to FR on KB cells or CHO-FRβ cells, and the relative affinity of FA for the FR was set to 1; that is, values <1 reflect weaker affinity than FA, and values >1 reflect stronger affinity.

### Dihydrofolate reductase inhibition assay

RAW264.7 cells growing in FFRPMI medium in 10 cm cell culture dishes (BD Falcon, Lincoln Park, NJ, USA) were treated with EC0746 (100 nM), EC0746 (100 nM) plus 100-fold molar excess of FA (10 μM), or FA alone (10 μM). After a 2-hour exposure, the drug-containing media were replaced and the cells were allowed to incubate further for 22 hours in fresh FFRPMI medium (referred in the text as a 22-hour chase). Meanwhile, AMT and MTX were kept at 100 nM for the entire 24-hour incubation period. All cells were subsequently lysed in the radioimmunoprecipitation assay lysis buffer, and DHFR activities in the whole cell lysates were determined using a commercial DHFR assay kit (Sigma-Aldrich). This spectrophotometric assay monitors the enzymatic conversion of dihydrofolic acid to tetrahydrofolic acid by DHFR and the disappearance of the co-factor nicotinamide adenine dinucleotide phosphate at 340 nm. The results were normalized to the values of the untreated control cells.

### XTT and TNFα assays

RAW264.7 cells in 96-well plates (3.5 × 10^4 ^cells/well) were treated with 10-fold serial dilutions of EC0746 (≤1 μM) in FFRPMI medium without and with 100-fold molar excess of FA. After a 2-hour exposure, the drug-containing media were replaced and the cells were allowed to incubate further for 70 hours (referred in the text as a 70-hour chase). In comparison, the cells were also treated continuously with AMT for 72 hours. Four hours prior to the end of incubation, LPS was added to the treated cells at a final concentration of 100 ng/ml. Then 100 μl culture supernatants were collected for TNFα analysis by ELISA. The cell viability was assessed by adding XTT to the remaining media for an additional 4 hours following the manufacturer's instructions. To evaluation of a cytostatic effect, RAW264.7 cells seeded at 1 × 10^6 ^cells/well in six-well plates were subjected to 2-hour exposure and a 70-hour chase with 0, 0.1, 10, and 1000 nM EC0746 without and with excess FA. At the end of the incubation (no LPS added), the surviving cells (that is, still viable cells) were recovered and redistributed in equal numbers in fresh medium for an additional 72 hours. The cell proliferation was again assessed by the XTT assay. All results were expressed as the percentage absorbance (minus background) relative to the untreated control cells.

### Rat cytokine array analysis

This analysis was performed on rat TG-macs to compare EC0746 against AMT and MTX for their abilities to inhibit cytokine production after LPS/IFNγ co-stimulation. Using our standard condition of 2-hour pulse plus a 70-hour chase period, rat TG-macs (harvested the day before) were given vehicle (media only), EC0746 (100 nM), EC0746 (100 nM) plus 100-fold molar excess of FA (10 μM), or FA alone (10 μM). For unconjugated base drugs, 100 nM AMT and MTX were present continuously for the entire 72-hour incubation period. Twenty-four hours prior to the end of incubation, LPS (5 μg/ml) and IFNγ (100 ng/ml) were added to the cells to stimulate cytokine production. The presence of cytokines/chemokines in the culture media was detected using a rat cytokine antibody array kit (R&D Systems) capable of detecting 29 analytes in duplicate spots. The total pixel intensity for each spot in the array was quantitated using the NIH ImageJ software [35], subtracted from the background, and averaged for each analyte.

### Adjuvant-induced arthritis

Prior to immunization with adjuvant, female Lewis rats were fed a folate-deficient diet (Harlan Teklad, Indianapolis, IN, USA) for ~10 days to reduce serum folate competition from high-folate-containing regular rodent chow [36]. The rats were then inoculated intradermally (at the base of tail) with 0.5 mg heat-killed *M. butyricum *(BD Diagnostic Systems) in 100 μl light mineral oil (Sigma-Aldrich, St Louis, MO, USA). Paw edema (degree of arthritis) in rats was assessed using an arthritis scoring system: 0 = no edema or arthritis; 1 = swelling in one type of joint; 2 = swelling in two types of joint; 3 = swelling in three types of joint; 4 = swelling of the entire paw [37]. A total score for each rat is calculated by summing the scores for each of the four paws, giving a maximum of 16 per animal. Notably, the first appearance of the signs or symptoms of arthritis in this model occurs around day 10 (typically between days 9 and 11) with distinctive but mild redness and/or swelling in small areas of the foot, but not necessary involving joints at that point. On the first day of treatment, rats with desired arthritis scores were distributed evenly across the control and treatment groups (*n *= 5). For each study, two or three rats from the same colony were not induced for arthritis and were used as healthy controls.

Unless noted otherwise, all drug treatments started on day 10 after arthritis induction and lasted for two consecutive weeks with biweekly (BIW, Mondays and Thursdays) or once-weekly (QW, Mondays) dosing regimens. At the completion of each study (day 24 or 4 days after the last treatment), rats were euthanized by CO_2 _asphyxiation and were processed for paw weight (cut at the hairline) and spleen weight. The removed hind paws were immersion-fixed in 10% buffered formalin and subjected to radiographic and/or histopathological analyses. When needed, X-ray radiographic images of the arthritic hind paws were taken using a Kodak Imaging Station In Vivo FX system (Carestream Molecular Imaging, New Haven, CT, USA).

EC0746 was given subcutaneously (s.c.) in a dosing range of 25 to 1,000 nmol/kg (QW or BIW); MTX was given s.c. or orally at 250 nmol/kg (BIW) or 1,650 nmol/kg (QW); and etanercept (10 mg/kg) was given s.c. once every 3 days for a 12-day span. The QW MTX dosing regimen was to mimic MTX administration in humans, and this particular dose of 1,650 nmol/kg per week (that is, 0.75 mg/kg per week) was reportedly active as an intraperitoneal agent in the AIA model [38]. To distinguish the anti-inflammatory mechanisms of EC0746 and MTX *in vivo*, a therapeutically irrelevant folate-containing competitor (EC0923, molecular weight 672) was used in 500-fold molar excess to block the activities of EC0746 and MTX at 250 nmol/kg (BIW).

### Radiographic and histopathological assessments

Formalin-fixed hind paws were examined by a board-certified veterinary radiologist who had no knowledge of the study groups. Specific criteria were used to establish a numerical grade of severity for each radiographic change: increased soft tissue volume (0 to 4), narrowing or widening of joint spaces (0 to 5), subchondral erosion (0 to 3), periosteal reaction (0 to 4), osteolysis (0 to 4), subluxation (0 to 3), and degenerative joint changes (0 to 3). Scores are limited to the tarsus, and the maximum possible score per foot was 26 [39]. The histopathological analysis was also performed in a blind fashion by an independent contract laboratory (Bolder BioPATH Inc., Boulder, CO, USA). The arthritic ankles were scored on a scale of 0 to 5 for inflammation, bone resorption, pannus formation, and cartilage damage, with a maximal histology score of 20 per foot [40].

### Pharmacokinetic studies

Female Lewis rats with jugular vein catheters (Harlan Sprague Dawley) were used to assess the plasma pharmacokinetics of EC0746 and unconjugated AMT. The animals were divided into two main groups, one given a single dose of EC0746 s.c. and the second a single dose of AMT s.c., both at 500 nmol/kg. Whole blood samples (300 μl) were collected from three animals per time point at the following time points: 1 minute, 10 minutes, 30 minutes, 1 hour, 2 hours, 3 hours, 4 hours, and 8 hours after injection. The blood samples were placed into anticoagulant tubes containing 1.7 mg/ml K_3_EDTA and 0.35 mg/ml *N*-maleoyl-β-alanine (0.35 mg/ml) in a 0.15% acetic acid solution. Plasma samples were obtained by centrifugation for 3 minutes at ~2,000 × *g *and stored at -80°C. The amounts of EC0746 and AMT in the plasma and the two primary metabolites of EC0746 (AMT and AMT hydrazide) were determined by liquid chromatography/mass spectrometry/mass spectrometry.

### Preliminary toxicity evaluations

The short-term toxicity and MTD of EC0746 and AMT were evaluated in healthy rats following the standard BIW subcutaneous dosing regimen used for efficacy studies. Further, these animals were put on a folate-deficient diet for ~20 days before treatment to match the folate deficiency status of AIA rats used for therapy. The folate-deficient but nonarthritic animals were thus given increasing doses of EC0746 and AMT for two consecutive weeks on a BIW basis. A MTD dose was defined as the dose that had caused at least 13 to 14% weight loss combined with clinical signs of stress, and at least one animal in the group receiving a dose greater than MTD needing to be euthanized. Standard hematologic and blood chemistry parameters were examined as needed along with histopathology.

### Statistics

Statistical analyses were performed using the computer program GraphPad Prism (GraphPad Software Inc., San Diego, CA, USA). Data were analyzed using Student's *t *test or the Mann-Whitney U test (nonparametric). If applicable, data were further analyzed across treatment groups using one-way analysis of variance. *P *< 0.05 was considered statistically significant in all tests.

## Results

### EC0746 folate receptor binding affinities

The chemical structure of EC0746 is shown in Figure 1a. There are four separate functional components to this novel construct: the FR-targeting moiety FA, the drug moiety AMT, a saccharo-amino acid peptide-based spacer of ((saccharo-γGlu)-γGlu)_2_-γCys, and a hydrazide/disulfide-containing linker. The sugar-modified peptide spacer has previously been shown to reduce the liver clearance of FA-drug conjugates [41,42]. The disulfide bond-based linker is designed to remain largely stable in the circulation but to fall apart quickly within the endosomal structures [43,44].

Like any FA-drug conjugate, the first step in the EC0746 screening process was to make sure that it maintains a high binding affinity towards the cell-surface FR to allow for efficient uptake via endocytosis. As shown in Figure 1b, EC0746 retains a relatively high binding affinity for both KB and CHO-FRβ cells with affinity values of 0.50 and 0.27, respectively. In contrast, AMT and MTX are both poor binders with their respective relative affinity values of 0.004 and 0.018 on KB cells and similar values of 0.004 and 0.005 on CHO-FRβ cells.

### Target-specific antiproliferative activity against RAW264.7 cells

**Figure 2:**
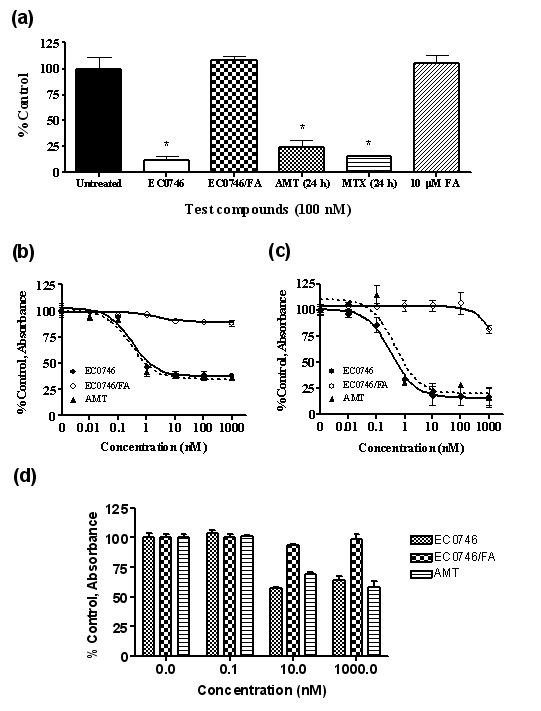
**EC0746 is a folate-receptor-specific dihydrofolate reductase inhibitor with potent cytostatic effect on RAW264.7 macrophages**. **(**a**)** RAW264.7 cells were given a 2-hour pulse of 100 nM EC0746 ± 10 μM folic acid (FA) followed by a 22-hour chase. Aminopterin (AMT) and methotrexate (MTX) were allowed to incubate for 24 hours. The dihydrofolate reductase activities in whole cell lysates (in duplicate) were normalized to untreated control cells (mean ± standard error of the mean). *P < 0.05. **(**b**), (**c**)** RAW264.7 cells were subjected to a 2-hour pulse followed by a 70-hour chase of a 10-fold serial dilution of EC0746 ± 100-fold molar excess of FA. Free AMT was allowed to incubate for 72 hours continuously. Four hours prior to the end of incubation, lipopolysaccharide (100 ng/ml) was added to stimulate TNFα production. The (b) cell viability and (c) TNFα in culture media were determined by XTT and ELISA assays, respectively. Results expressed as the percentage of control in absorbance (mean ± standard error of the mean in triplicates). **(**d**)** RAW264.7 cells were treated with indicated concentrations of EC0746 ± excess FA (2-hour pulse plus a 70-hour chase). The surviving cells were redistributed in equal numbers in fresh medium and allowed to incubate further for 72 hours. The cell proliferation was again determined by the XTT assay.

AMT is a potent inhibitor of DHFR, and therefore we tested the ability of EC0746 to inhibit DHFR in a manner that was dependent on its cell uptake by FR-mediated endocytosis. For this purpose, we employed a FR-positive subclone of the murine macrophage-derived RAW264.7 cell line (see Materials and methods). The parent RAW264.7 macrophage cell line has been widely used for the study of immunosuppressive drugs [45], and in our opinion could serve as a model for a subpopulation of inflammatory monocytes and macrophages that have proliferative capacity [46,47]. Hence, FR-positive RAW264.7 cells were given only a 2-hour pulse of 100 nM EC0746 without or with a 100-fold excess of FA (10 μM) followed by a 22-hour chase. In comparison, the cells were also treated with 100 nM AMT and MTX, except that these untargeted drugs were left on cells for the entire 24-hour incubation period. As shown in Figure 2a, EC0746 activity was similar to AMT and MTX with regard to the extent of DHFR inhibition; however, the inhibitory activity of EC0746 was blocked by excess FA, indicating that the observed DHFR inhibition was dependent on FR-mediated cellular uptake. Notably, RAW264.7 cells were 100% viable under these treatment conditions and whole cell lysates were recovered for the determination of DHFR activity. As an additional control, exposure of the cells to FA alone was found to be benign.

Because DHFR is an enzyme that is critical for the S-phase of cell proliferation [48], EC0746 was evaluated for its antiproliferative activity in comparison with AMT. RAW264.7 cells (at ~40% confluency) were exposed for 2 hours to 10-fold serial dilutions of EC0746 (0.01 nM to 1 μM) without or with 100-fold excess FA, followed by a 70-hour chase. In addition, LPS (100 ng/ml) was added to the culture media 4 hours before the end of incubation to stimulate the release of TNFα, a key proinflammatory product of activated macrophages. Meanwhile, RAW264.7 cells were treated with AMT for 72 hours continuously over the same concentration range. As determined by the XTT assay (Figure 2b), EC0746 showed a dose-dependent inhibition of cell proliferation with a relative 50% inhibitory concentration value of ~0.3 nM. Importantly, the observed antiproliferative effect was 100% competitive in the presence of excess FA, indicating a FR-specific mode of action. Likewise, EC0746-treated RAW264.7 cells produced less TNFα after LPS stimulation with a relative 50% inhibitory concentration value of ~1.6 nM, and the observed effect was also 100% competitive by excess FA (Figure 2c). Interestingly, EC0746 appeared to have a cytostatic effect on RAW264.7 cells with a maximum growth inhibition of ~50% at concentrations ≥1 nM. In fact, these surviving cells could no longer divide when redispersed into fresh medium for an additional 72-hour incubation (Figure 2d).

Taken together, these data demonstrate that EC0746 completely halted the proliferation of RAW264.7 cells in a FR-dependent manner but did not kill them; instead, these cells appeared to have experienced a prolonged arrest. The reason for such comparison is that, following the initial 2-hour pulse, the majority of EC0746 will remain bound to the cell surface FR for subsequent internalization during the drug-free chase period, whereas the untargeted AMT will not bound.

### Immunomodulatory effect on rat peritoneal macrophages

**Figure 3:**
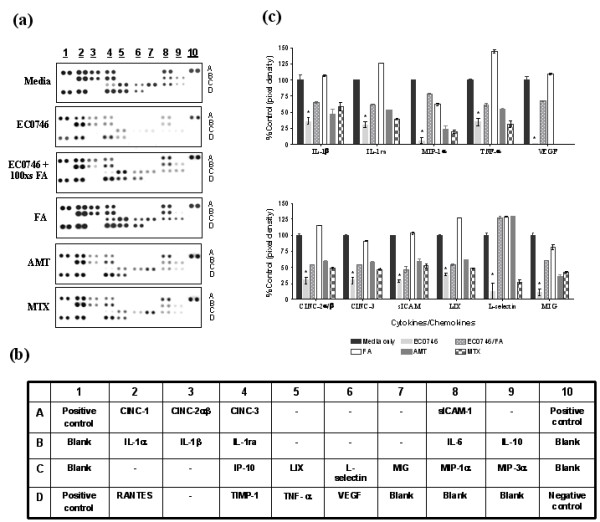
**EC0746 has an immunomodulatory effect on folate-receptor-expressing rat TG-macs**. Rat TG-macs were treated with media only, 100 nM EC0746 ± 10 μM folic acid (FA), or FA alone (10 μM) for 2 hours followed by a 70-hour chase. In comparison, the cells were also treated with 100 nM free aminopterin (AMT) and methotrexate (MTX) for 72 hours. At 24 hours before the end of incubation, all cells were stimulated with lipopolysaccharide (LPS) (5 μg/ml) plus IFNγ (100 ng/ml). The cytokines/chemokines produced in culture supernatants were detected using a rat cytokine array. **(**a**)** Cytokine release profiles of rat TG-macs stimulated with LPS and IFNγ with or without drug treatment. **(**b**)** Cytokine array map. **(**c**)** Mean pixel intensity (*y *axis) determined for each array position and plotted for the 11 products, which were detected at three times above background levels and in at least three independent experiments. Data shown are mean ± standard error of the mean. *P < 0.05 when compared with its corresponding cytokine level in the media only sample. CINC, cytokine-induced neutrophil chemoattractant; LIX, LPS-induced CXC chemokine; MIG, monokine induced by IFNγ; MIP-1α, macrophage inflammatory protein-1α; RANTES, regulated upon activation, normal T-cell expressed and secreted; sICAM, soluble intracellular adhesion molecule; TIMP-1, tissue inhibitor of metalloproteinase 1; VEGF, vascular endothelial growth factor.

Unlike RAW264.7 cells, rat TG-macs display little proliferative activity *ex vivo *and therefore were used in our studies to represent inflammatory macrophages in a low proliferative state. Not surprisingly, neither EC0746 nor AMT or MTX affected TG-macs viability after 72-hour incubation at concentrations as high as 10 μM. As TG-macs can be further activated *in vitro*, however, we explored the ability of EC0746 to block cytokine production after exposing them to LPS and IFNγ, two signals required for a full activation of macrophages [49]. Using our standard condition of a 2-hour pulse with the test article plus a 70-hour chase, TG-macs were treated with 100 nM EC0746 without or with excess FA for competition. LPS (5 μg/ml) and IFNγ (100 ng/ml) were then added to the treated cells 24 hours prior to the end of incubation to stimulate the release of cytokines/chemokines, which were then detected with a rat cytokine antibody array. As shown in Figure 3a,b, LPS/IFNγ co-stimulation of TG-macs promoted the release of ~19 cytokines/chemokines, 11 of which (Figure 3c) showed a FR-specific inhibition by EC0746 (in at least three independent analyses), including a few key proinflammatory mediators (TNFα, IL-1β, macrophage inflammatory protein-1α, monokine induced by IFNγ, and so forth). These data collectively indicated that the levels of FRs on TG-macs were sufficient for EC0746 to remedially affect cytokine responses associated with macrophage activation, and that the observed anti-inflammatory action of EC0746 can be independent of anti-macrophage proliferation.

### Assessment of efficacy and dose/schedule dependency *in vivo*

To establish a proof of concept for EC0746 *in vivo*, we chose the macrophage-rich rat AIA model where a preferential uptake of FA-targeted imaging agents is consistently seen in sites of active inflammation (arthritic paws, liver, and spleen) [25,26]. The rat AIA model resembles many characteristics of RA in humans and has been widely used for the study of novel anti-inflammatory agents [50]. In our animal studies (*n *= 5 per group), the onset of arthritis usually occurred around day 10 after intradermal inoculation of *M. butyricum *and was very aggressive. Multiple study endpoints were taken to assess the effectiveness, including the arthritis score (that is, paw edema) measured by a semiquantitative visual scoring system (see Materials and Methods), the change in body weight (at the plateau of the disease, untreated control animals lost ~14 to 20% of their original weights), the paw weight, as an alternative assessment of paw edema, and the spleen weight, as an assessment of splenomegaly (an enlargement of the spleen).

In a preliminary study (data not shown), a BIW regimen of EC0746 (500 nmol/kg, s.c.) was tested in AIA rats presenting with varying degrees of arthritis (for example, mean arthritis scores of ~0 versus 2 on the first day of treatment). EC0746 was found to be fast acting in treating AIA from the disease onset (that is, mean starting arthritis score of ~0 on day 10); consequently, these animals maintained a low arthritis score (~1) and a steady body weight throughout the course of study. In rats with more established diseases (for example, mean starting arthritis score of ~2 on days 10 to 13 post induction), EC0746 treatment also improved the overall severity of the disease, but to a lesser extent. Here, the maximum reduction in arthritic scores was ~50%, but the accompanying weight loss due to the induction process was not reversed. When the percentage increases in paw and spleen weights were analyzed, EC0746 treatment yielded ~10-fold (paw edema) and threefold (splenomegaly) improvements in rats with low starting arthritis, and the corresponding improvements were ~2.5-fold and twofold in rats bearing more established diseases.

To fully investigate the dose-response relationship and schedule dependency, EC0746 was administered s.c. to rats starting around the disease onset (days 9 to 11; mean, day 10) with a dose range of 25 to 500 nmol/kg BIW, or 1,000 nmol/kg given QW. As summarized in Table 1, EC0746 treatment on days 10, 13, 17, and 20 displayed an ~10-fold linear dose response from 25 to 250 nmol/kg, with *R*^2 ^values of 1.00 (percentage inhibition in paw edema) and 0.99 (reduction in splenomegaly), respectively. The maximal activity of EC0746 was achieved at 250 nmol/kg per dose, yielding ~91% inhibition in paw edema, >3-fold to fourfold improvement in splenomegaly, and with no apparent weight loss. There was no statistical difference between the 250 and 500 nmol/kg dosing regimens of EC0746 in all endpoints assessed, suggesting a (FR) saturating dose response in efficacy. When administered QW at 1,000 nmol/kg (days 10 and 17), EC0746 remained effective with ~72% inhibition in paw edema, but this schedule did not control the fast progressing AIA to the same degree as the optimal BIW dosing regimen (≥250 nmol/kg). Because of the schedule-dependent nature of the response, animals receiving the QW EC0746 treatment also lost ~7% of their original body weights due to arthritis progression (Table 1). In summary, EC0746 was shown to be highly effective against AIA, more effective when dosed BIW than QW, and capable of halting disease progression by controlling both local (joints) and systemic (spleen) inflammation.

**Table 1:** EC0746 anti-arthritis activity in comparison with methotrexate and etanercept

Treatment	Dose	Frequency	Inhibition in paw edema (%)^a^	Splenomegaly^b^	Body weight change (%)^c^
Control	-	-	0	117 ± 22	-16 ± 1
EC0746 (s.c.)	25 nmol/kg	Biweekly	0 ± 25^d^	73 ± 8^e^	-17 ± 1
	100 nmol/kg	Biweekly	35 ± 11^d^	52 ± 13^e^	-14 ± 1
	250 nmol/kg	Biweekly	91 ± 4^d^	25 ± 6^e^	-0.5 ± 3
	500 nmol/kg	Biweekly	91 ± 9	37 ± 7	0.4 ± 4
	1,000 nmol/kg	Once weekly	72 ± 12	39 ± 5	-7 ± 3
MTX (p.o.)	250 nmol/kg	Biweekly	70 ± 5	42 ± 17	-14 ± 2
	1,650 nmol/kg	Once weekly	47 ± 10^f^	-	-10 ± 2
MTX (s.c.)	250 nmol/kg	Biweekly	78 ± 10	24 ± 3	-2 ± 4
	1,650 nmol/kg	Once weekly	63 ± 13^f^	-	-7 ± 8
Etanercept (s.c.)	10 mg/kg	Every 3 days	46 ± 9^f^	42 ± 7	-15 ± 2

Rats with adjuvant arthritis were treated at disease onset (10 days post arthritis induction) with EC0746, methotrexate (MTX), and etanercept at indicated doses, dosing routes, and dosing frequencies. s.c., subcutaneously; p.o., per orally. ^a^Inhibition in paw edema is calculated based on paw weight on day 24: 100 × (arthritic control - treated)/(arthritic control - healthy). ^b^Splenomegaly is defined as the percentage increase in spleen weight relative to the spleen weights of healthy rats. ^c^On day 24 relative to body weight on the first day of treatment (day 10). Linear regression analysis: ^d^*R*^2 ^= 1.00 (paw) and ^e^*R*^2 ^= 0.99 (spleen). ^f^Calculated based on arthritis scores on day 24 (paw weights were not obtained).

### *In vivo *folate receptor specificity: proof of concept

**Figure 4:**
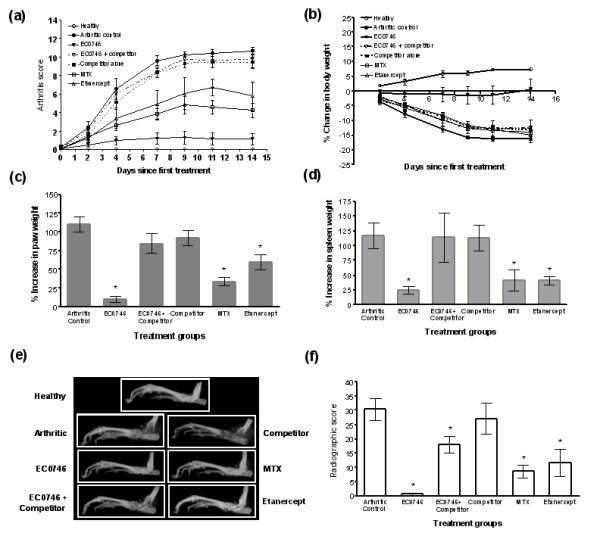
**EC0746 demonstrates folate-receptor-specific anti-inflammatory activities *in vivo***. Starting on day 10 after arthritis induction, rats with developing adjuvant-induced arthritis (*n *= 5) were given a biweekly subcutaneous dosing regimen of EC0746 (250 nmol/kg) without or with a 500-fold molar excess of EC0923 as the folate competitor. For comparison purposes, methotrexate (MTX) (250 nmol/kg) was dosed orally following the same schedule as EC0746, and etanercept (10 mg/kg) was given subcutaneously on days 10, 13, 16, 19, and 22. Multiple endpoints are shown: **(**a**)** arthritis score; **(**b**)** change in body weight; **(**c**) **percentage increase in paw weight; **(**d**)** percentage increase in spleen weight; **(**e**)** representative X-ray images of arthritic hind paws taken using a Kodak Imaging Station; and **(**f**)** radiographic score of arthritic hind paws. *P < 0.05 when compared with the arthritic control group.

**Figure 5:**
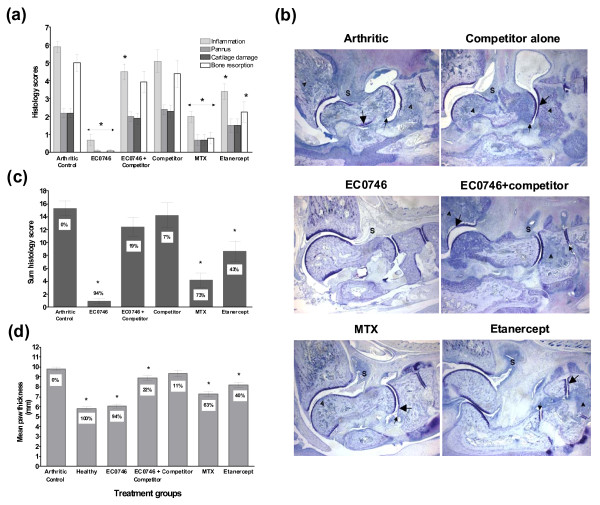
**Histopathological assessment**. The histopathological analysis was performed on formalin-fixed arthritic hind paws by Bolder BioPATH Inc. (Boulder, CO, USA). **(**a**)** Individual histological scores of ankle joints on a scale of 0 to 5 for inflammation, bone resorption, pannus formation, and cartilage damage with a maximal histology score of 20 per foot. **(**b**)** Representative photomicrographs (16x) of the ankle closest to the **(c) **mean summed histological score. **(**d**)** Dorsal to ventral paw thickness for each group. Notably, the arthritic control animal showed very severe inflammation (S), bone resorption (arrowhead) with mild pannus (small arrow), and cartilage damage (large arrow). *P < 0.05 when compared with the arthritic control group. MTX, methotrexate.

To confirm *in vivo *target specificity of EC0746, we conducted competition studies in AIA rats using a benign folate-containing competitor (EC0923) to block the FR binding advantage of EC0746 (Figures 4 and 5). EC0923 (pteroyl-γGlu-d-Asp-d-Asp) is a high-affinity water-soluble FA-peptide conjugate used in our laboratory for *in vivo *competition studies rather than FA because high doses of the latter can cause renal damage due to precipitation in the kidneys [51]. As described in Materials and methods, four groups of AIA rats were given a standard BIW subcutaneous dosing regimen of either nothing (that is, arthritic control), EC0746 alone (250 nmol/kg), EC0746 (250 nmol/kg) plus a 500-fold molar excess of EC0923 (125 μmol/kg), or EC0923 alone (125 μmol/kg). All treatments lasted for 2 weeks, beginning 10 days after the arthritis induction (disease onset).

As illustrated in Figure 4, EC0923 alone did not have any impact on the development or severity of arthritis. EC0746 alone was highly effective, as expected from previous results (Table 1). Conversely, the activity of EC0746 was nearly completely blocked by the presence of co-administered EC0923 in all clinical parameters assessed: arthritis score (Figure 4a), change in body weight (Figure 4b), and percentage increases in paw (Figure 4c) and spleen weights (Figure 4d). Radiographic analysis of the arthritic paws (Figure 4e,f) confirmed minimal radiographic changes in EC0746-treated animals (similar to the healthy controls), whereas significant joint erosions were seen in the untreated arthritic controls and in the animals that had been treated with EC0923 alone or with EC0746 plus EC0923.

Microscopically (Figure 5a,b), severe joint deteriorations (that is, synovial inflammation, bone resorption, pannus formation, and cartilage damage) were detected in the arthritic control animals and in the animals treated with EC0923. In contrast, three out of five animals treated with EC0746 had no lesions, resulting in 88 to 100% decreases in individually scored parameters (Figure 5a), thus representing an overall decrease of 94% in the summed scores (Figure 5c). Notably, the animals treated with EC0746 plus the 500-fold excess of EC0923 had a significantly decreased inflammation score (24%; Figure 5a), but all other scored parameters were nonsignificantly decreased (9 to 21%; Figure 5a). Accordingly, the EC0746/EC0923-treated arthritic animals had an overall decrease of ~19% in the summed scores, which was significantly less than the 94% reduction in animals treated with EC0746 alone (*P *< 0.05; Figure 5c). Likewise, the dorsal to ventral paw thickness in the EC0746/EC0923-treated animals was decreased by 22%, far less than the 94% reduction in the EC0746-treated animals (*P *< 0.05; Figure 5d).

Overall, the results presented in Figures 4 and 5 show a good correlation between macroscopic and microscopic examinations of the arthritic animals, supporting the fact that the anti-arthritic activities of EC0746 were predominantly FR mediated.

To better understand EC0746 specificity *in vivo*, we turned our attention to AMT and MTX. Both agents are active comparators of EC0746, the former being the parent drug and the latter being the most commonly prescribed antifolate in the clinic. Given the large differences in FR affinities (Figure 1b) and the abilities of MTX and AMT to enter other cells via RFC or protein-coupled folate transporter, EC0746 was predicted to affect a different population of host immune cells than MTX and AMT, especially in a situation where FR-positive macrophages play a big role in chronic inflammatory responses. For years, RA patients receiving antifolate therapy have been given folate supplementation to reduce adverse effects and to extend treatment durations [12,52]. Because EC0746 contains both FA and AMT moieties, a question arose as to whether the anti-arthritic activity of EC0746 in AIA rats was due to apparent folate supplementation of AMT. In an effort to address this question, we mixed unmodified AMT with FA (1:1) and dosed AIA rats at a level matching the well-tolerated BIW dose of EC0746 (500 nmol/kg s.c.). Unfortunately, after one or two doses, all animals treated with the simple mixture had to be euthanized due to severe anemia and gastrointestinal distress (lethargy, bloody diarrhea, and so forth). More importantly, whereas AMT is obviously a very toxic agent, its FA-targeted form (that is, EC0746) is not.

**Figure 6:**
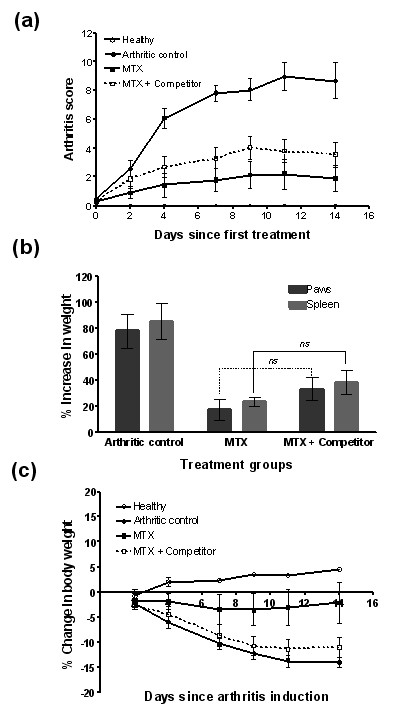
**Methotrexate displays a lack of competition for folate-receptor-binding sites and a nonfolate-receptor-targeted anti-arthritic activity**. In an identical dosing fashion as was carried out for EC0746 in Figure 4, adjuvant-induced arthritis rats (*n *= 5) were treated subcutaneously with methotrexate (MTX) (250 nmol/kg biweekly) for 2 weeks without or with a 500-fold molar excess of EC0923 as the folate competitor. Anti-arthritic activities are shown: **(**a**)** arthritis score; **(**b**) **percentage increases in paw and spleen weights; and **(**c**)** percentage change in body weight. ns, not significant.

Regarding MTX, which is a weaker FR binder than EC0746, one might predict that the activity of MTX in AIA rats would not be blocked by EC0923 under the same competing conditions described above. To investigate this hypothesis, three separate groups of AIA rats were s.c. dosed BIW with either nothing, MTX alone (250 nmol/kg), or MTX (250 nmol/kg) plus excess EC0923 (125 μmol/kg). As assessed by arthritis scores (Figure 6a), percentage increases in paw and spleen weights (Figure 6b), and the change in body weight (Figure 6c), the anti-arthritic activity of MTX was not significantly blocked by the presence of the EC0923 competitor (*P *> 0.05; see figure legends). Taken together, these data confirmed that EC0746 and MTX were different from each other with regards to treating active inflammation via FR-targeted and non-targeted mechanisms of action, respectively.

### EC0746 is more efficacious than oral methotrexate and subcutaneous etanercept

Since MTX and etanercept are part of the current standard of care for RA, we compared EC0746 against both drugs in the rat AIA model using clinically relevant dosing routes. In people, MTX is generally taken QW by mouth, but there is a considerable variation in bioavailability (28 to 94% at 15 mg/week) [53]. In our preliminary tests, we found QW oral MTX treatment (0.75 mg/kg or 1,650 nmol/kg) yielded ~50% efficacy in AIA rats (based on arthritic scores), but the animals experienced ~10% weight loss due to disease progression (Table 1). This was not unexpected as MTX also has a low and variable oral bioavailability in rats due to limited absorption and intestinal degradation (21% at 0.1 mg/kg) [54]. On an equimolar basis, QW MTX (1,650 nmol/kg) s.c. was found to be slightly more potent than QW oral MTX, yielding ~63% maximum reduction in arthritis score and a slightly less weight loss (~7%) (Table 1). Conversely, etanercept is a fully humanized recombinant TNF receptor (p75)-Fc fusion protein given as BIW injections to patients who have failed or have become less responsive to MTX [5]. Returning to our study, shown in Figures 4 and 5, EC0746 was also compared directly against oral MTX on an equimolar BIW basis (that is, 250 nmol/kg), while etanercept (10 mg/kg) was s.c. dosed every 3 days over a 12-day span (see Materials and methods). Although rodents develop neutralizing antibodies against etanercept, this limited high-dose regimen of etanercept was reported to be active in rodent models of arthritis [55,56].

As expected, both MTX and etanercept were active in improving various symptoms of the experimental AIA (Figure 4 and Table 1), but their effects were far from optimum and neither therapy controlled arthritis quickly enough to prevent substantial weight loss (Figure 4b and Table 1). Histological grading of arthritic ankles (Figure 5) showed oral MTX-treated animals had significant reductions (66 to 84% from the untreated arthritic controls) in all scored parameters (Figure 5a,b), and there was a 73% significant decrease in the summed score (Figure 5c). While etanercept did not appear to be as effective as oral MTX, animals treated with etanercept also had significant reductions in inflammation (42%) and bone resorption (55%), which contributed to a significant 43% decrease in the summed score (Figure 5a,c). Further, the dorsal to ventral paw thicknesses in both MTX-treated and etanercept-treated AIA rats were significantly decreased by 63% and 40%, respectively (Figure 5d). In almost all parameters assessed (Figures 4 and 5, and Table 1), however, s.c. administered EC0746 consistently outperformed both oral MTX and etanercept regimens - showing greater improvements in arthritis scores, arthritis-related weight loss, paw edema, radiographic changes, histological scores, and dorsal to ventral paw thicknesses. Notably, all three agents decreased splenomegaly in AIA rats, but their effects were not statistically different from each other (Table 1, calculated from Figure 4d).

### Pharmacokinetics and metabolism

As shown in Table 1, EC0746 demonstrated a linear dose-efficacy relationship in AIA rats, suggesting good bioavailability after administration s.c. Because EC0746 contains a hydrazide/disulfide-based releasable linker (Figure 1a), we anticipated that AMT and AMT hydrazide would be the two primary metabolites *in vivo*. On a cellular level, AMT and AMT hydrazide were found to be equally potent in the inhibition of cell proliferation and LPS-stimulated TNFα production in RAW264.7 macrophages (data not shown). To study the pharmacokinetics of EC0746, healthy rats were given a single subcutaneous injection of EC0746 (500 nmol/kg) and the plasma concentrations of EC0746 as well as the potential metabolites, AMT, and AMT hydrazide, were monitored for up to 8 hours. For comparison, unconjugated AMT was examined at a matching dose (500 nmol/kg s.c.) after a single administration.

EC0746 was found to reach the bloodstream within minutes, with the maximum concentration (321 nmol/l) occurring approximately 10 to 30 minutes post dose, and maintained a plateau until 60 minutes after the injection. EC0746-derived AMT and AMT hydrazide were both detectable in plasma with maximum concentration values of 23 and 11 nmol/l, respectively (curves nearly superimposable), but both metabolites showed an approximate 30-minute delay from the time at which EC0746 maximum concentration occurred. While EC0746 itself was cleared rapidly from the blood with an elimination half-life of ~35 minutes, the elimination half-lives of the two metabolites were three to five times longer at 117 minutes (AMT) and 187 minutes (AMT hydrazide), respectively. The corresponding area under the curve values for EC0746 and its metabolites AMT and AMT hydrazide were 32.5, 4.4, and 2.9 nmol*minute/ml. Similar to EC0746, the maximum concentration (601 nmol/l) of unconjugated AMT in the plasma occurred ~30 minutes after dosing; however, its elimination half-life was ~140 minutes, which is actually comparable with the values for EC0746-derived AMT and AMT hydrazide. The area under the curve value of the s.c. dosed AMT was measured at 61.3 nmol*minute/ml.

Taken together, EC0746 metabolism *in vivo *appeared to result in a delayed release of AMT and AMT hydrazide, but these two metabolites behaved more like free AMT rather than EC0746 with regards to elimination. Based on the area under the curve responses, ~18% of active drug exposure/release (AMT plus AMT hydrazide) was detected in the plasma over the 8-hour collection period in the EC0746-dosed animals.

### Preliminary short-term toxicity assessment

Since the toxicity of antifolates can be easily masked by rodent diets enriched with FA [57], healthy rats on a folate-deficient diet (Harlan Teklad, Madison, WI, USA) were used to determine the MTDs of EC0746 and AMT. Following the same dosing regimen used for aforementioned anti-arthritis therapies (that is, four subcutaneous doses given in 2 weeks), the MTDs of EC0746 and AMT were determined to be 2,000 nmol/kg and 50 nmol/kg, respectively, representing a 40-fold difference in toxicity. At supra-MTD doses, the toxicologic findings in EC0746-treated rats were similar to those treated with AMT, including diarrhea, swollen muzzle, leucopenia, thrombocytopenia, and opportunistic infections. While immunosuppression and gastrointestinal toxicity appear to be dose-limiting for these compounds in folate-deficient rats, EC0746 showed less of the gastrointestinal-associated side effects than AMT at their respective MTD doses.

## Discussion

There are two natural isoforms of the membrane FR (FRα and FRβ), and both bind FA with a high affinity (*K*_D _<1 nM) [58]. FRα is best known for its overexpression on human epithelial cancers, and is present on the apical surfaces of limited normal epithelial cell types (proximal tubules of the kidneys, choroid plexus of the brain, and alveolar epithelial cells of the lungs) [58]. FRβ is found on myelogenous leukemias [59] and has now become a promising biomarker for activated macrophages and monocytes [21-23]. To date, it is not entirely clear which subpopulation(s) of activated macrophages (and monocytes) expresses FRβ and what role these receptors play in chronic inflammation [24]. Adding to this complexity, activated macrophages are known to be a heterogeneous population of extraordinarily versatile cells that are functionally dynamic based on their microenvironment [49]. Because FR-positive macrophages are found to express various macrophage activation markers (that is, CD80, CD86, Ly6C/G, TNFα, and reactive oxygen species) [22], depleting or inactivating this effector cell population may have a profound effect on the immune system. In the present study we evaluated EC0746, a novel FA-AMT construct that utilizes the high-affinity FA ligand as a targeting moiety to facilitate the specific delivery of an anti-folate pharmacophore to FRα/FRβ-expressing cells (Figure 1b) and to inhibit DHFR in a FR-dependent manner (Figure 2a). Notably, our relative affinity values for AMT and MTX are consistent with previously reports for these and other antifolates that have weak affinities for both FR isoforms because their preferred route of cellular entry is via the RFC [19].

Since it was challenging to find an *in vitro *cell model that would realistically mimic the pathophysiological properties of activated macrophages *in vivo*, we employed the high FR-expressing and highly proliferative RAW264.7 macrophages, as well as the low FR-expressing and low-proliferating rat TG-macs, to represent macrophages in different states of activation/inflammation. Rat TG-macs were chosen because they could be easily obtained, and they express a functional FR at a similar level to that of peritoneal macrophages isolated from AIA rats at the plateau of their disease [25]. Moreover, the rat TG-macs isolated as described represent a heterogeneous population of ~90% CD11b/c-positive cells, and ~70% of the CD11b/c-positive cell population are FR-positive upon staining with a fluorescent folate probe (data not shown). Using these two cell models, EC0746 treatment resulted in two independent but FR-specific mechanisms of action: an anti-proliferative effect against RAW264.7 cells (Figure 2b to 2d), and an anti-inflammatory effect against rat TG-macs without affecting cell viability or proliferation (Figure 3). Overall, our *in vitro *results suggested that EC0746 might be active against a heterogeneous population of FR-positive macrophages (and monocytes) at sites of active inflammation. It remains uncertain, however, whether macrophages from the peritoneal cavity differ from those present in an inflamed joint, whether rat TG-macs have other immunoinflammatory mechanisms that make them different from macrophages isolated from the arthritic animals, and whether *ex vivo *isolated macrophages lose FR expression and, consequently, inflammatory impetus in cell culture.

For *in vivo *efficacy assessment, the rat AIA model has been our choice due to its well-documented systemic inflammation involving activated macrophages [60,61] and the presence of FR-positive macrophage subpopulations [25]. The obvious disadvantage of this model is its aggressive and acute nature (full-blown arthritis in 2 to 3 weeks), which makes it unlike human RA. Owing to the difficulty of obtaining relevant clinical samples, we have not been able to directly compare the level of FR expression on activated macrophages in AIA rats with that found on cells within the joints of RA patients. A second, cautious limitation of this model is that the circulating serum folate levels in rats are supra-physiologically high due to the supplementation of commercial rodent chows [36]. Because unnaturally high folate levels can act as a competitor for FR binding, rats used in this study were fed a nonsupplemented diet. Although speculative, it is possible that FR levels in resident macrophages may have inadvertently been upregulated in the arthritic rats during the course of study. Nonetheless, BIW EC0746 treatment (s.c.) was found to be highly effective in alleviating overall symptoms of AIA, especially when the treatment started at disease onset. The EC0746 anti-arthritic activity was also dose and schedule dependent (Table 1), and appeared to be FR-specific since a benign folate ligand (EC0923) could efficiently block its overall effect by competing for FR-binding sites *in vivo *(Figures 4 and 5). Although MTX is a weak bicarboxylic acid structurally related to FA, its binding affinities to FR-expressing cells (KB, CHO-FRβ) were ~56-fold to 200-fold lower than that of FA (Figure 1b). Accordingly, the anti-arthritic activity of MTX was not blocked to a significant degree by EC0923 under the same competing conditions (Figure 6). This finding also supported EC0923 compromising the efficacy of EC0746 through its interaction with the FR and not through any antifolate mechanism of action. Finally, as a structure-matched negative control, we also tested an analog of EC0746 that was constructed with the unnatural, and biologically inert d-enantiomer of AMT. This compound, which shares similar FR binding affinity and identical molecule weight to EC0746, was found to be completely inactive both *in vitro *and *in vivo *(data not shown). These results helped confirm that an anti-inflammatory response is not automatically triggered by the simple binding of a ligand to the FR present on activated macrophages, and that specific activity is dependent on the endocytosis of the conjugate followed by biological cleavage of the linker and cytosolic release of a biologically active drug.

MTX generally appears as effective as EC0746 on an equimolar basis when both drugs are administered BIW via a subcutaneous route in AIA rats (Table 1). Our *in vitro *and *in vivo *results, however, confirmed that these two agents were significantly different from each other with regards to targeting FR-positive and FR-negative inflammatory cells. In that regard, Matsuyama and colleagues - who studied MTX transport via FRβ in RA synovial macrophages - suggested that folate antagonists with a higher affinity towards FRβ could be more useful in RA treatment [21]. EC0746 did consistently outperform oral MTX on an equimolar basis, and it appeared to be more active than etanercept (Figures 4 and 5, and Table 1), although the latter is a genetically engineered human protein with unknown potency to rat TNFα. Limited by its molecular properties (size, charge, hydrogen bonding potential, and so forth), EC0746 does not meet the common criteria for oral drug delivery [62], so this route of dosing was not initially explored. In addition, the high toxicity of AMT in rodents precluded further investigation into the anti-arthritic activity of this base drug. In fact, we found in folate-deficient rats that EC0746 was approximately 40-fold less toxic than AMT (all given by subcutaneous administration). Folate conjugation and targeting therefore provided a therapeutic window to AMT where none exists otherwise in AIA rats. It is worth mentioning, however, that AMT is currently being studied in psoriasis patients using very low doses (NCT00937027; Syntrix Biosystems Inc., Auburn, WA, USA).

Pharmacokinetically, EC0746 (500 nmol/kg) administered s.c. was absorbed quickly into the central compartment from the subcutaneous space, with the time to maximum concentration occurring at approximately 10 to 30 minutes, followed by a short elimination half-life (~35 minutes). In comparison, unconjugated AMT at the matching subcutaneous dose exhibited a similar time to maximum concentration of 30 minutes, but the elimination half-life increased significantly (~140 minutes). Based on our experiences with folate-targeted chemotherapeutic agents, a shorter elimination half-life (as seen for EC0746) is favorable because it minimizes nonspecific tissue exposure without reducing the FR targeting potential [42]. EC0746 was also found to be metabolized *in vivo*, and the two major metabolites (AMT and AMT hydrazide) exhibited longer elimination half-lives than EC0746. Because antifolates like AMT enter normal cells via the RFC or protein-coupled folate transporter, we suspect that EC0746-derived AMT/AMT hydrazide would behave like free AMT on a cellular level (except for a 30-minute delay in appearance in the plasma). A comparison of the area under the curve values for EC0746 and released AMT/AMT hydrazide indicates that as much as ~18% free drug was detected in the plasma. The question therefore arose regarding how much these two metabolites might contribute to the overall efficacy and toxicity of EC0746 administered to AIA rats. While this topic deserves further study, there are three reasons we do not believe that free drug significantly affected the potency of EC0746 therapy. First, we have shown that a 2-week BIW treatment of 500 nmol/kg EC0746 s.c. is a safe and effective dosing regimen (Table 1). Using the pharmacokinetic observations as a guide for exposure at this dose level, and considering the fact that roughly 18% free drug is released in circulation following a 500 nmol/kg EC0746 dose, then a 90 nmol/kg dose of AMT would be predicted to be both effective and nontoxic. Not only did our toxicology studies show that doses of AMT above 50 nmol/kg resulted in considerable morbidity and mortality, however, but 40-fold higher EC0746 doses were found to be well tolerated. Second, the anti-arthritic activity of EC0746 could be blocked by the presence of the folate competitor EC0923 (Figures 4 and 5), indicating that the mechanism of action for EC0746 was specific for FR expression and not a nontargeted effect due to the metabolites AMT/AMT hydrazide. Third, animal data presented within this report suggest that the efficacy of EC0746 was probably not driven entirely by its pharmacokinetic properties in the plasma, but rather by its dynamics on target cells expressing a functional FR.

Targeting activated macrophages via the FR is a rational approach to curtail macrophages' resistance to apoptosis and to inhibit their ability to produce tissue-damaging products. Our investigation provides the first evidence that anti-inflammatory drugs, like AMT, can be directly linked to FA or folate-like ligands to yield an improved therapeutic index. Recently, a few orally active small molecule drugs have shown promising results in MTX-failure patients, including two Janus kinase inhibitors, INCB028050 [50] and CP-690,550 [63,64], and the Syk kinase inhibitor R788 [65]. These novel anti-inflammatory agents appear to be better tolerated and they can yield similar efficacy as anti-TNF biologics. As most rheumatologists still prefer MTX as the first disease-modifying anti-rheumatic drug for RA treatment, if approved these new agents are likely to position themselves after MTX failure but before the use of biologics. Owing to the lack of MTX-resistant animal models of inflammation, the ability of EC0746 to overcome MTX resistance was not investigated. In the clinic, studies have shown the need to gradually increase the dose of MTX in order to achieve an optimal therapeutic benefit, and some people will eventually become nonresponsive to MTX. One of the reasons for the lack of response is that RFC-mediated transport across the cell membrane utilizes an anion exchange mechanism that requires a higher saturating dose to work efficiently [66]. EC0746 may overcome such deficiency by delivering AMT to FR-positive inflammatory macrophages and increasing their intracellular retention of the drug.

## Conclusions

The therapeutic value of EC0746 needs to be validated in the clinic compared against other existing treatment options. One encouraging fact may be that inflammatory macrophages and monocytes do not turnover as fast as their normal counterparts, and these cells may maintain their FR expression in time to allow for FR-specific intervention. For long-term therapy and management, EC0746 would probably cost less to produce than biologics, and it could potentially be self-administered at a frequency yet to be determined in the clinic (not in animal models). Overall, the potential application of EC0746 may include macrophage-rich inflammatory disorders such as RA, psoriasis, atherosclerosis, Crohn's disease, uveitis, vasculitis, and diabetes (many of which share some common pathogenic mechanisms).

## Abbreviations

AIA: adjuvant-induced arthritis; AMT: aminopterin; BIW: biweekly; DHFR: dihydrofolate reductase; ELISA: enzyme-linked immunosorbent assay; FA: folic acid; FFRPMI: folate-free RPMI 1640 medium; FR: folate receptor; IFN: interferon; IL: interleukin; LPS: lipopolysaccharide; MTD: maximum tolerated dose; MTX: methotrexate; QW: once weekly; RA: rheumatoid arthritis; RFC: reduced-folate carrier; s.c.: subcutaneously; TG: thioglycollate; TNF: tumor necrosis factor; XTT: 2,3-bis(2-methoxy-4-nitro-5-sulfo-phenyl)-2H-tetrazolium-5-carboxanilide.

## Competing interests

Except for PSL, all authors are full-time employees and stockholders of Endocyte, Inc. PSL is a full-time professor at Purdue University (West Lafayette, IN, USA). PSL is also a founder, the Chief Science Officer, and a compensated member of Endocyte's Board of Directors. A patent application has been submitted to cover the data disclosed in this manuscript. No other financial interests apply for any authors.

## Authors' contributions

YL contributed to the experimental design, data acquisition, data analysis and interpretation, and drafted the manuscript. TWS contributed to the *in vitro *experimental design, data acquisition, and data analysis and interpretation. EW and VAC contributed to *in vivo *data acquisition and data analysis. PJK and MAG contributed to design, and data analysis and interpretation involving toxicology and pharmacokinetics. IRV contributed to synthetic chemistry involving EC0746 and EC0923. PSL contributed to experimental design and edited the manuscript. CPL contributed to the experimental design, data analysis and interpretation, and edited the manuscript. All authors read and approved the final manuscript.
